# Supplementary material for: Dynalign II: common secondary structure prediction for RNA homologs with domain insertions
Source: Nucleic Acids Res. 2014 Nov 21;42(22):13939–48. doi: 10.1093/nar/gku1172 (PMC4267632; doi:10.1093/nar/gku1172)
Supplement: SUPPLEMENTARY DATA [file supp_gku1172_nar-02021-z-2014-File012.zip › manual/Text/ProbabilityPlot.html]

RNAstructure Command Line Help: ProbabilityPlot


|  |  |  |
| --- | --- | --- |
|  | RNAstructure Command Line Help ProbabilityPlot | - Contents - Index |
| ProbabilityPlot is used to create a base pairing probabilities plot after calculating the partition function (which can be done using the partition interface). USAGE: ProbabilityPlot <input file> <output file> [options] Required parameters: <input file> \| The name of the input file that holds base pairing probabilities.; <output file> \| The name of a file to which output will be written. Depending on the options selected, this may be one of the following file types. 1) A Postscript image file. 2) An SVG image file. 3) A plain text file. Options which don't require added values: -h, -H, --help \| Display the usage details message.; --svg \| Specify that the output file should be an SVG image file, rather than a Postscript image file.; -t, -T, --text \| Write dot plot as a text file, rather than a Postscript image file. Options which require added values: -e, -E, --entries \| Specify the number of categories in the plot legend. Default is 5 entries.; -max, -MAX, --maximum \| Specify the maximum value in the plot in kcal/mol. Default is the largest valid value in the plot.; -min, -MIN, --minimum \| Specify the minimum value in the plot in kcal/mol. Default is the smallest valid value in the plot. References: 1. Reuter, J.S. and Mathews, D.H. "RNAstructure: software for RNA secondary structure prediction and analysis." *BMC Bioinformatics*, 11:129. (2010). 2. Mathews, D.H. "Using an RNA secondary structure partition function to determine confidence in base pairs predicted by free energy minimization." *RNA*, 10:1178-1190. (2004). 3. McCaskill, J.S. "The equilibrium partition function and base pair probabilities for RNA secondary structure." *Biopolymers*, 29:1105-1119. (1990). | | |
| Visit The Mathews Lab RNAstructure Page for updates and latest information. | | |
